# Cyclic fatigue resistance of EdgeTaper Platinum, Protaper Gold, and TruNatomy Prime rotary files before and after autoclave sterilization

**DOI:** 10.7717/peerj.14656

**Published:** 2023-01-20

**Authors:** Rahaf A. Almohareb, Reem M. Barakat, Fahda N. Algahtani, Manal F. Alkadi

**Affiliations:** Department of Clinical Dental Sciences, College of Dentistry, Princess Nourah Bint Abdulrahman University, Riyadh, Saudi Arabia

**Keywords:** Autoclave, Cyclic fatigue, Edgetaper platinum, Trunatomy, Heat-treated file, Nickel-titanium, Rotary files

## Abstract

**Background:**

This *in vitro* investigation aimed to determine the influence of multiple autoclave cycles on the cyclic fatigue resistance of three heat-treated nickel-titanium (NiTi) files: EdgeTaper Platinum (ETP), ProTaper Gold (PTG), and TruNatomy Prime (TN).

**Materials:**

Sixty NiTi files, twenty of each NiTi file type: ETP 25/.06, PTG 25/.08, and TN 26/.04 were randomly divided into four equal subgroups (*n* = 5). The files for the control group were left un-autoclaved. Different autoclave sterilization cycles (one, five, and ten) were used for the other three groups. The files were then placed in a metal canal block and rotated according to the manufacturer’s instructions until fracture. The length of the broken segment and the time taken for fracture were measured. The fractured surfaces were subsequently subjected to SEM imaging. The Kruskal-Wallis test was used to analyze the data, followed by Dunn-Bonferroni pairwise comparisons. Statistical significance was set at *p* < 0.05.

**Results:**

ETP showed significantly greater resistance to cyclic fatigue than TN in all autoclave groups and PTG after five autoclave cycles (*p* = 0.014). Fatigue resistance was not affected by the number of autoclaving cycles, except for ETP. After the first and tenth autoclaving cycles, they required significantly more rotations to failure than the non-sterilized files (*p* = 0.039 and *p* = 0.021, respectively). The fractured segments of the ETP files in these two groups were also longer than those in the control group (*p* = 0.010).

**Conclusion:**

The cyclic fatigue resistance of ETP was greater than that of TN in all tested conditions. Repeated autoclave cycles of sterilization improved the cyclic fatigue resistance of the ETP files only and did not affect the cyclic fatigue resistance of TN and PTG. However, the ETP files separated at a longer distance from the tip with increased autoclaving cycles.

## Introduction

The introduction of nickel-titanium alloy (NiTi) in the production of dental root canal files ushered in a new era of endodontics, as these files reduced the incidence of treatment-related mishaps (Alrahabi, 2017). However, endodontic NiTi rotary files can fracture inside root canals during endodontic treatment. Fractured instruments may irreparably block access to diseased root canal regions and adversely affect the outcomes of endodontic treatment (Siqueira, 2001).

The incidence of NiTi file fractures increases in complex root canal systems with acute or abrupt curvature (Ferreira et al., 2017). The two causes of NiTi file fractures are cyclic fatigue and torsional failure (Setzer & Böhme, 2013). However, cyclic fatigue is considered the primary cause of NiTi file fractures in curved root canal systems (Shen et al., 2009). Therefore, the metallurgic and mechanical properties of NiTi rotary files are constantly being improved to enhance their cyclic fatigue resistance (Haapasalo & Shen, 2013). Instrument-related characteristics such as alloy composition, design, and manufacturing processes have been modified (Haapasalo & Shen, 2013). Other factors that could potentially impact NiTi file resistance to fracture include the number of uses, endodontic irrigation solutions, operating kinetics, vertical magnitude, axial stresses, and rotating speed (Haapasalo & Shen, 2013).

NiTi instrument sterilization procedures involve thermomechanical processing and extra heat treatment. This can greatly influence their shape memory and super-elastic capabilities (Craveiro De Melo, De Azevedo Bahia & Buono, 2002). However, these findings are highly dependent on NiTi file metallurgy, design, and manufacturing processes (Plotino et al., 2012). Autoclave cycles reportedly have no adverse effects on or can potentially reduce the cyclic fatigue resistance of some NiTi files (Hilfer et al., 2011; Kim et al., 2020).

The EdgeTaper Platinum (ETP) file is a new edition of the EdgeTaper (ET) rotary file that has undergone heat treatment to improve its mechanical properties (EdgeTaper, Albuquerque, NM, USA). The manufacturer claims that this edition has superior cyclic fatigue resistance properties when compared to ET and ProTaper Gold (PTG) files (EdgeTaper, Albuquerque, NM, USA). On the other hand, the TruNatomy Prime (TN) file is a heat-treated file that was recently released and has a smaller cross-section and taper (Riyahi et al., 2020). Rotary files with larger cross-sections may be more prone to fracture in challenging cases (Wu et al., 2011), while small files may show high incidences of deformation and their single use has been recommended. (Fernández-Pazos et al., 2018).

Previous studies have compared the impact of serial autoclave sterilization on the cyclic fatigue resistance of PTG *vs.* ETP files (Phumpatrakom et al., 2022) and TN *vs.* PTG files (Venkatesh & Varghese, 2022). However, no studies have compared the three NiTi systems, and more specifically TN and ETP, in the same experiment. Rotary file comparisons are only reliable when they are performed under the same experimental conditions (Plotino et al., 2009). Therefore, the objective of this randomized controlled investigation was to compare the cyclic fatigue resistance of three heat-treated rotary files, ETP, PTG, and TN, before and after autoclave sterilization. The null hypothesis stated that there are no differences in cyclic fatigue resistance among the three thermally treated files before and after multiple autoclave cycles.

## Materials & Methods

The study was registered at the Dental College of Princess Nourah Bint Abdulrahman University in Riyadh, Saudi Arabia and was exempt from ethical approval by the Institutional Review Board (21-0482).

## Materials

Three new types of thermally treated NiTi rotary files were tested in this study: the ETP 25/.06 file (EdgeEndo, Albuquerque, NM, USA), the PTG 25/.08 file (Dentsply Sirona, Ballaigues, Switzerland), and the TN 26/.04 file (Dentsply Sirona, Maillefer, Ballaigues, Switzerland). Table 1 presents the manufacturers’ descriptions of each of these files. Sample size calculations were performed using G*Power 3.1 software (Heinrich-Heine-Universität, Düsseldorf, Germany). A total of 60 files were used to obtain results with 80% power and a type I error rate (*α*) of 0.05.

**Table 1:** Manufacturers’ descriptions of the NiTi files.

**NiTi file**	**Tip diameter**	**Length**	**Taper**	**Cross-section**	**NiTi Wire**
**EdgeTaper Platinum**	0.25 mm	25 mm	Progressively variable 0.06	Bloated triangular	Heat-treated Firewire (1.2 mm diameter)
**TruNatomy**	0.26 mm	25 mm	Regressively variable 0.04	Convex triangular	Thin heat-treated wire (0.8-mm diameter)
**ProTaper Gold**	0.25mm	25 mm	Progressively variable 0.08	Off-center parallelogram	Heat-treated wire (1.2-mm diameter)

### Autoclaving procedure

**Figure 1:**
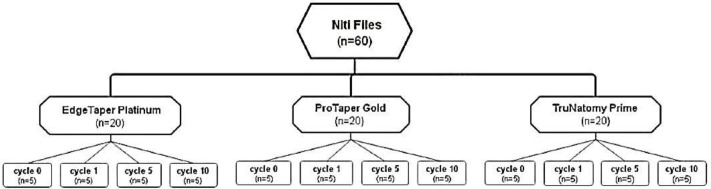
Distribution of the sample into different experimental groups according to number of autoclave sterilization cycles.

Figure 1 shows the distribution of the sample into the different experimental groups. Twenty files of each NiTi file type (ETP 25/.06, PTG 25/.08, and TN 26/.04) were randomly selected. Each group was randomly divided into four equal subgroups (*n* = 5). Group (cycle 0) served as the control group with files left without autoclave sterilization. The other three groups were treated with different numbers of autoclave sterilization cycles (one, five, and 10 cycles) (Ba-Hattab et al., 2022). During each cycle, the files were sterilized for 4 min at 132 °C under 29 psi pressure in a steam sterilizer (Steris Amsco Century Prevac Steam Sterilizer V148 h, USA) and then dried for 5 min (Almohareb et al., 2021).

### Cyclic fatigue testing

**Figure 2:**
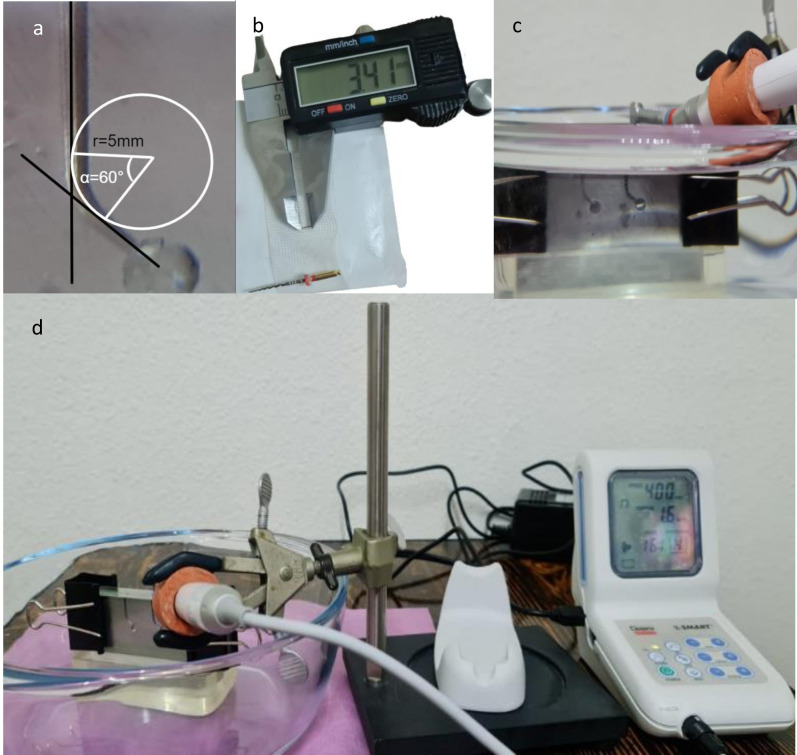
The cyclic fatigue experiment setting. (A) The artificial canal model with angle and radius of curvature readings. (B) Measuring device for measuring the length of fractured file. (C) The rotary file inserted inside the artificial canal that is immersed in water bath. (D) An overview of the experiment setting.

All experimental groups underwent cyclic fatigue resistance testing using a method recommended by previous studies (Plotino et al., 2012; Almohareb et al., 2021). This method employed a customized stainless-steel block in which a canal with a 60-degree curve, 5-mm radius, 1.40-mm inner diameter, and 19-mm length was constructed (Fig. 2A).

A 16:1 reduction dental handpiece (X-Smart; Dentsply Sirona) attached to an endodontic electric motor was mounted on a pre-constructed jig that enabled precise, repetitive insertion of the NiTi files into the artificial canal. The metal block was submerged in a 35 °C water bath to simulate body temperature. (Figs. 2C and 2D). The NiTi files were then placed in the canal and rotated in a continuous clockwise manner until they broke. All of the files were operated using the torques and speeds recommended by the manufacturer. For ETP and PTG files, we used a rotational speed of 300 RPM and a torque of 3 N-cm, whereas for TN, we used a speed of 500 RPM and a torque of 1.5 N-cm. Instrument separation was observed visually and aurally. One operator blinded to the file types measured the time required for fracture in seconds using an electronic timer. The number of cycles required for fracture was calculated accordingly: number of cycles for fracture = rotation speed’ time to fracture. The lengths of the separated segments were also measured in millimeters (Fig. 2B).

### Scanning electron microscopy (SEM)

After separation, the fractured surfaces of the files were inspected and their topographical properties were evaluated using SEM (JSM-7001F; JEOL, Tokyo, Japan). Photomicrographs of the shattered surfaces were obtained at ×250 and ×2000 magnifications, along with images of the lateral surfaces at ×100 magnification.

### Statistical analysis

SPSS Statistics for Windows, version 22.0 (IBM Corp., Armonk, NY, USA) was used for data analysis. After normality was assessed with the Shapiro–Wilk test, data were analyzed with the Kruskal–Wallis test followed by the Dunn–Bonferroni pairwise comparison test. Statistical significance was set at *p* < 0.05.

## Results

**Figure 3:**
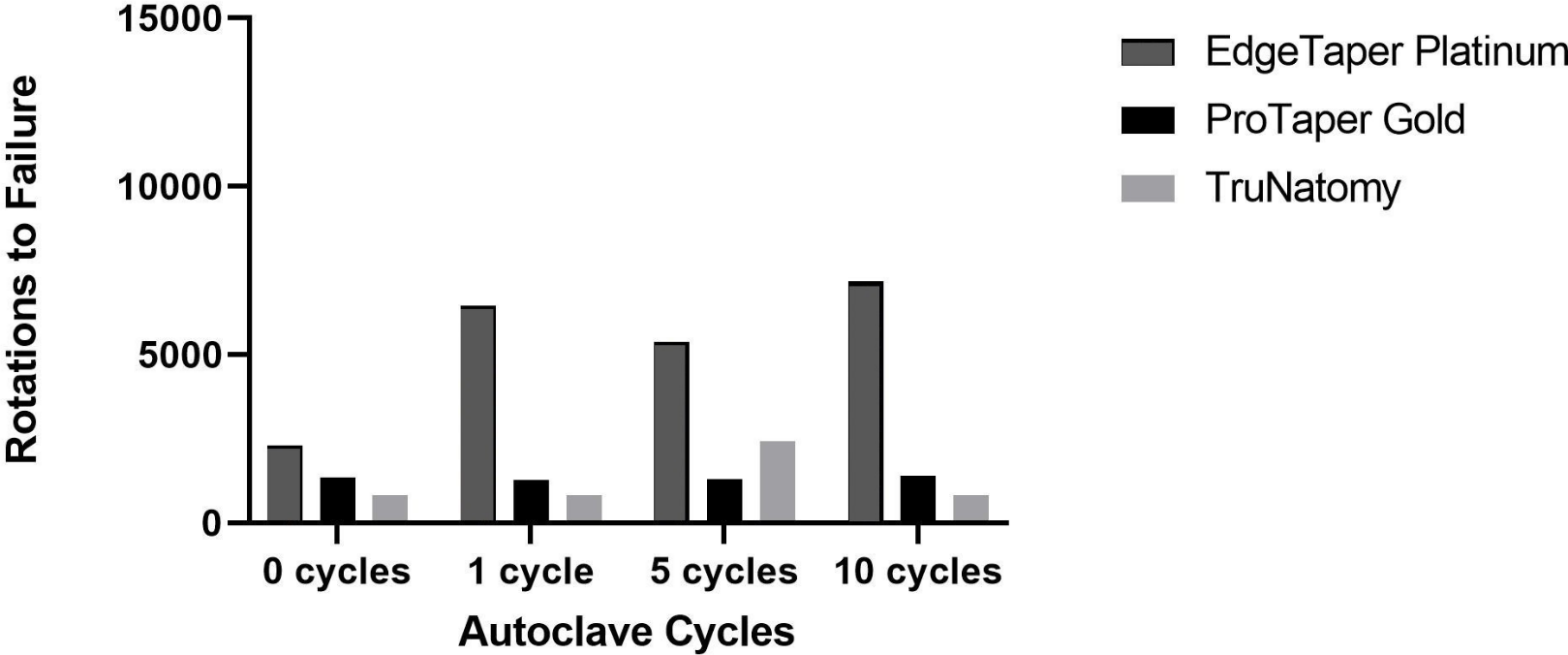
Number of rotations to file failures before and after autoclaving one, five and 10 cycles for the three file systems.

The number of rotations to failure for ETP, PTG, and TN files before (the control group) and after autoclaving are presented in Fig. 3. There were significant differences between the three NiTi files in terms of the number of rotations to failure. ETP files showed significantly more resistance to cyclic fatigue than TN files irrespective of the number of autoclave cycles (Table 2) and PTG files after five autoclave cycles (*p* = 0.014).

**Table 2:** Non-parametric comparisons of the three NiTi file types in terms of the number of rotations to failure and lengths of fractured segments before and after autoclaving.

**NiTi files**		**EdgeTaper Platinum**	**ProTaper Gold**	**TruNatomy**	***p*-** **value**
**Number of autoclave cycles**		**Mean rank**	**Mean rank**	**Mean rank**	
**0**	Rotations	8.00^a^	5.00	2.00^b^	0.027^*^
	Segment length (mm)	5.33	3.67	6.00	0.561
**1**	Rotations	13.00^a^	6.60	4.40^b^	0.007^*^
	Segment length (mm)	12.60^a^	3.00^b^	8.40	0.003^*^
**5**	Rotations	13.00^a^	5.00^b^	6.00^b^	0.009^*^
	Segment length (mm)	12.60^a^	5.20^b^	6.20	0.018^*^
**10**	Rotations	13.00^a^	6.80	4.20^b^	0.006^*^
	Segment length (mm)	11.60	7.00	5.40	0.075

**Notes.**

a,b Different letters indicate significant differences between the values.

* Level of significance set at *p* < 0.05.

Fatigue resistance was not affected by the number of autoclave cycles, with the exception of ETP files (Table 3). After the first and tenth autoclave cycles, the EPT files showed more resistance to cyclic fatigue than the non-autoclaved files (*p* = 0.039 and *p* = 0.021, respectively). The fractured segments of the files in these two groups were also longer than the non-autoclaved files (control group) (*p* = 0.0010). Thus, the null hypothesis was rejected for ETP files.

**Table 3:** Number of rotations to fracture and lengths of fracture segments for each NiTi file type before and after autoclaving.

**NiTi files**	**Sterilization cycles**	**Rotations to failure**		**Length of fractured segments**	
		**Mean rank**	***p*-** **value**	**Mean rank**	***p*-** **value**
**EdgeTaper Platinum**	0	2.00^a^	0.010^*^	3.17^a^	0.030^*^
		1		12.60^b^			13.20^b^	
		5		7.00			7.00	
	10	13.40^b^		12.10^b^	
**ProTaper Gold**	0	11.50	0.742	9.17	0.726
		1		9.00			7.50	
		5		7.70			11.30	
	10	10.60		9.90	
**TruNatomy Prime**	0	8.33	0.239	13.33	0.723
		1		8.40			11.70	
		5		11.80			6.90	
	10	9.00		13.33	

**Notes.**

a,b Different letters indicate the presence of significant differences.

* Level of significance set at *p* < 0.05.

An analysis of the lengths of fractured segments also revealed that the fractured segments of the ETP files were considerably longer than those of the PTG files after the first and fifth autoclave cycles (*p* = 0.002 and *p* = 0.026, respectively). However, no significant differences were observed between the PTG and TN files (Table 2).

**Figure 4:**
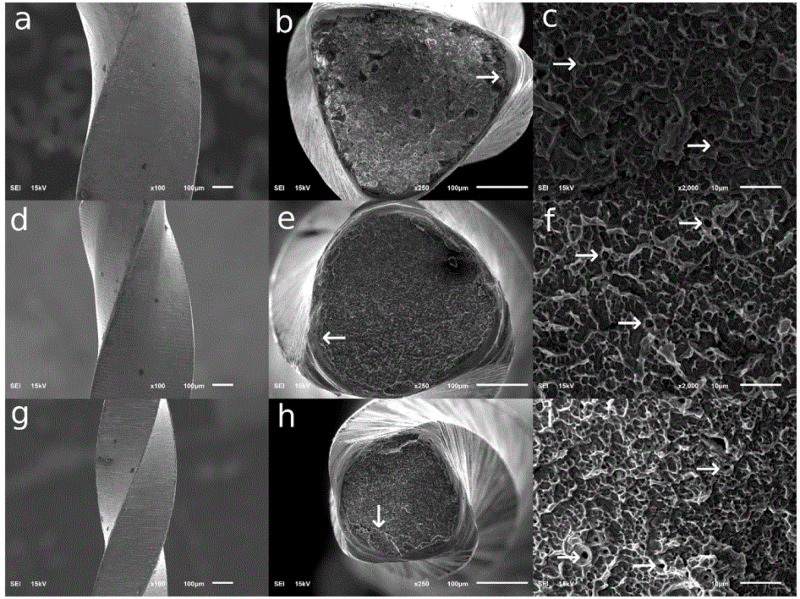
Scanning electron microscope photomicrographs of the fractured surfaces after 10 cycles. (A–C) EdgeTaper Platinum; (D–F) ProTaper Gold; (G–I) TruNatomy Prime.

Figure 4 shows SEM photomicrographs of the fractured surfaces of the three NiTi file types after 10 autoclave cycles. The arrows indicate distinct features associated with ductile fractures such as dimpling, micro-voids, and small crack initiation surfaces.

## Discussion

Numerous new NiTi rotary systems have recently been introduced for root canal instrumentation, with the objective of expanding instrument lifespan, and minimizing undesirable events such as intracanal instrument separation that may negatively affect treatment outcomes. Many of these new systems include manufacturing process modifications such as the application of special thermal treatments to optimize the NiTi alloy microstructure. To maximize the benefit of the improved lifespans by reusing these instruments multiple times, autoclave sterilization procedures are essential. However, these procedures may also affect the instruments’ physical and mechanical properties (Dioguardi et al., 2021). Therefore, this study evaluated the effect of multiple autoclave cycles on the cyclic fatigue resistance of three newly developed heat-treated NiTi files (ETP, PTG, and TN).

Our findings showed that autoclave sterilization did not affect the cyclic fatigue resistance of the PTG or NT files. ETP files, on the other hand, exhibited greater sensitivity to sterilization procedures and became more resistant to cyclic fatigue failure. Resistance to cyclic fatigue after both one and ten autoclave cycles increased compared to non-autoclaved files. A previous study reported no effect of autoclave sterilization on ETP files. However, in agreement with our findings, PTG files were also unaffected (Phumpatrakom et al., 2022). In contrast, another study showed increased resistance to cyclic fatigue in PTG files after 10 autoclave sterilization cycles (Özyürek, Yılmaz & Uslu, 2017). These discrepancies could be due to the different methodologies implemented in each study. A recent systematic review of the effects of autoclave sterilization on the cyclic fatigue resistance of heat-treated instruments revealed high investigation method heterogeneity and a general lack of standardization, in turn impairing meaningful comparisons between different studies and/or the formulation of evidence-based conclusions (Silva et al., 2020). Another systematic review similarly reported conflicting findings; however, the authors concluded that NiTi files exhibit a trend toward improved cyclic fatigue resistance following autoclave sterilization (Dioguardi et al., 2021).

Although the tested files had similar tip sizes and lengths, their different tapers should be taken into consideration when interpreting the results. Previous studies have shown that reduced instrument taper and core mass improve cyclic fatigue resistance (Faus-Llácer et al., 2021). Therefore, this study intended to examine this phenomenon in three heat treated files which had different tapering. Indeed, the ETP files, which have a smaller taper (0.06) than PTG files (0.08), showed significantly higher cyclic fatigue resistance after five sterilization cycles. This observation was consistent with that of a previous study (Phumpatrakom et al., 2022). However, an alternative explanation for this finding may be the differing heat treatments to which the two file types may have been subjected. Different thermal processing methods can produce different Austenite finishing (Af) temperatures. It has been shown that high Af temperatures are associated with the presence of a desirable martensitic phase at body temperature during endodontic treatment (Jamleh et al., 2019). Although TN files have a smaller taper, which generally leads to better cyclic fatigue resistance, ETP files exhibited significantly higher resistance to cyclic fatigue than TN files (Faus-Llácer et al., 2021). Other factors may have an influence in this context, including different file geometries such as longitudinal and cross-sectional designs. It has been reported that a cross-sectional design, rather than instrument size or taper, has a greater influence on the stress generated on an instrument under a load (Zhang, Cheung & Zheng, 2010). Additionally, the rotational speed and torque at which files are operated have also been reported to affect cyclic fatigue resistance (Pedullà et al., 2014). In the present study, TN files were operated at higher speeds and lower torques than ETP files (500 *vs.* 300 RPM and 1.5 *vs.* 3 N-cm, respectively); these parameters were selected according to the manufacturers’ recommendations. Increased rotational speed has been shown to negatively affect cyclic fatigue resistance (Pérez-Higueras, Arias & De la Macorra, 2013). This phenomenon was attributed to the higher rates of stress and strain generated with increasing RPMs, as well as the associated limited amount of time for instrument relief (Martín et al., 2003).

Interestingly, in the present study, we observed that the lengths of the fractured fragments of the ETP files increased in parallel with the increase in cyclic fatigue resistance. In other words, as the number of cycles before fracture increased, the instrument separated at a point far from the instrument tip where the cross-sectional diameter of the file was larger. Previous studies have shown that instruments with larger sizes (larger cross-sectional diameters) are more susceptible to cyclic fatigue than smaller instruments (Adıgüzel & Capar, 2017). This result was attributed to the larger effect of compression and tension stresses generated on the external surface of the file, as that surface is located farther away from the central axis where the effects of these stresses are minimized or negated (Faus-Llácer et al., 2021). This phenomenon may also explain the findings observed in our study regarding the shift of the ETP file fracture location to a point with a larger cross-section as the cyclic fatigue resistance increased. As the file became more resistant to cyclic fatigue, areas with smaller cross-sections may have become more resistant, whereas regions with larger cross-sections may have been more prone to cyclic fracture. It is also possible that autoclaving influences the mechanical properties of the files at points with different cross-sectional diameters. Indeed, it has been reported that torsional failure resistance and cutting efficiency decline in heat-treated files as their flexibility increases (Silva et al., 2019). Thus, torsional failure may result in longer file fractures as was observed with ETP files.

SEM image analysis is typically used to confirm the type of fracture an instrument has endured. Fractographic analysis is also an essential step for excluding the presence of other types of fractures such as torsional failure. The SEM images from our study showed that all three files had similar characteristics indicating ductile separation due to flexural fatigue: an initiation point surrounded by a surface exhibiting dimpling and microvoid coalescence. Similar findings have been reported previously (Phumpatrakom et al., 2022). Additionally, a study of different heat-treated PTG and ETP files described similar fracture surface details but with more distinct striation lines (Almohareb et al., 2021). Generally, ductile fracture is an uncommon type of material failure but occurs when a material has endured severe stress (Hayes, Edwards & Shah, 2015). Therefore, clinicians must be aware of the negative impact of curved root canals on the life expectancies of rotary files. Specifically, the position and radius of the canal curvature are important parameters that affect the levels of stress and strain, and thus cyclic fatigue, to which NiTi files are subjected; higher stress levels are produced with decreasing curvature radii (Necchi et al., 2008). In the present study, canal curvature was standardized at 60 degrees with a 5-mm radius. Further studies are needed to see how these files behave when subjected to higher stress levels such as in canals with small radii of curvature and/or different curvature locations (Lopes et al., 2011).

The current study has some limitations. First, the static cyclic fatigue test used did not include dynamic axial movements that would better simulate clinical conditions. These movements mimic the picking motions of the file in the root canal and have been reported to increase cyclic fatigue resistance owing to the different localizations of the points of maximum curvature/stress along the instrument as it moves vertically. In addition, in clinical situations, instruments are subjected to torsional load and cyclic fatigue simultaneously. When combined, these types of stresses may affect instruments’ physical and mechanical properties differently. Furthermore, in clinical contexts, instruments undergo sterilization procedures after use, the latter of which also affects their lifespans. However, the effect of clinical use was not evaluated in this study. Thus, further investigations that mimic the entire clinical scenario should be performed to better understand the combined effects of canal instrumentation and autoclaving. A final aspect of our study that limits its clinical relevance is the fact that the tested files rotated in contact with metal which is different from dentin. Unfortunately, it is not possible to accurately standardize the canal curvature of natural teeth; moreover, NiTi files rotating in natural teeth will always be subjected to a combination of torsional and cyclic fatigue stresses. Hence, testing was conducted in a metallic canal in order to better control these variables and avoid biases (Lopes et al., 2011).

The clinical implications of this study can be summarized as follows: All three file types had comparable cyclic fatigue resistances when tested under standardized extreme conditions. However, the null hypothesis was rejected in the present study: First cyclic fatigue resistance of ETP files was superior to that of TN files. Second, autoclave sterilization had a positive impact on the cyclic fatigue resistance of ETP files. While this could help assure clinicians that the procedure is safe, the effects of sterilization on other physical properties of rotary files such as cutting efficiency and resistance to torsional failure need to be explored in future studies. Moreover, the clinical incidence of ETP, PTG, and TN file separation was not studied. These clinical studies are needed to address the performance of these files in real-life scenarios and assess risks associated with file separations. Not to mention the need to explore the role of the operator. Experienced operators had fewer incidences of file separation and procedural mishaps (Yared, Bou Dagher & Machtou, 2001). Therefore, sufficient training is essential to ensure the safe operation of rotary instruments.

## Conclusions

Despite the limitations of this study, ETP rotary files exhibited greater resistance to cyclic fatigue failure than TN files. ETP files also had greater resistance to cyclic fatigue failure than PTG rotary files after five cycles of autoclave sterilization. Moreover, repeated autoclave cycles increased the cyclic fatigue resistance of ETP files but did not affect that of TN or PTG files. However, the ETP files also separated at a longer distance from the tip with increasing numbers of autoclave cycles. Future studies are needed to study the effects of sterilization on the cutting efficacy and torsional failure resistance of these files.

##  Supplemental Information

10.7717/peerj.14656/supp-1 Supplemental Information 1 Results

10.7717/peerj.14656/supp-2 Data S1 Raw data

## Additional Information and Declarations

### Data Availability

The following information was supplied regarding data availability: The raw data is available in the Supplemental Files.

## References

[ref-1] Adıgüzel M, Capar ID (2017). Comparison of cyclic fatigue resistance of waveone and waveone gold small, primary, and large instruments. Journal of Endodontics.

[ref-2] Almohareb RA, Barakat R, Albakri A, Altamimi M (2021). Effect of autoclaving cycles on the cyclic fatigue resistance of race and race evo nickel-titanium endodontic rotary files: an *in vitro* study. Metals.

[ref-3] Alrahabi M (2017). Capacità di diversi sistemi in nichel-titanio nella preparazione di canali artificiali con doppia curvatura. Giornale Italiano di Endodonzia.

[ref-4] Ba-Hattab R, Almohareb RA, Alkhalaf R, Binnjefan S, Sulayem M, Barakat RM (2022). The impact of multiple autoclave cycles on the surface roughness of thermally treated nickel-titanium endodontic files. Advances in Materials Science and Engineering.

[ref-5] Craveiro De Melo MC, De Azevedo Bahia MG, Buono VTL (2002). Fatigue resistance of engine-driven rotary nickel-titanium endodontic instruments. Journal of Endodontics.

[ref-6] Dioguardi M, Arena C, Sovereto D, Aiuto R, Laino L, Illuzzi G, Laneve E, Raddato B, Caponio VCA, Dioguardi A, Zhurakivska K, Troiano G, Muzio LL (2021). Influence of sterilization procedures on the physical and mechanical properties of rotating endodontic instruments: a systematic review and network meta-analysis. Frontiers in Bioscience-Landmark.

[ref-7] Faus-Llácer V, Hamoud-Kharrat N, Marhuenda Ramos MT, Faus-Matoses I, Zubizarreta-Macho Á, Ruiz Sánchez C, Faus-Matoses V (2021). Influence of the geometrical cross-section design on the dynamic cyclic fatigue resistance of NiTi endodontic rotary files—an *in vitro* study. Journal of Clinical Medicine.

[ref-8] Fernández-Pazos G, Martín-Biedma B, Varela-Patiño P, Ruíz-Piñón M, Castelo-Baz P (2018). Fracture and deformation of ProTaper Next instruments after clinical use. Journal of Clinical and Experimental Dentistry.

[ref-9] Ferreira F, Adeodato C, Barbosa I, Aboud L, Scelza P, Zaccaro Scelza M (2017). Movement kinematics and cyclic fatigue of NiTi rotary instruments: a systematic review. International Endodontic Journal.

[ref-10] Haapasalo M, Shen Y (2013). Evolution of nickel-titanium instruments: from past to future. Endodontic Topics.

[ref-11] Hayes MD, Edwards DB, Shah AR (2015). Fractography in failure analysis of polymers: a volume in plastics design library.

[ref-12] Hilfer PB, Bergeron BE, Mayerchak MJ, Roberts HW, Jeansonne BG (2011). Multiple autoclave cycle effects on cyclic fatigue of nickel-titanium rotary files produced by new manufacturing methods. Journal of Endodontics.

[ref-13] Jamleh A, Alghaihab A, Alfadley A, Alfawaz H, Alqedairi A, Alfouzan K (2019). Cyclic fatigue and torsional failure of edgetaper platinum endodontic files at simulated body temperature. Journal of Endodontics.

[ref-14] Kim W, Oh S, Ryu GJ, Kim TH, Kim SJ, Kim DH, Lee BN, Kum KY, Chang SW, Jang JH (2020). Effect of autoclave sterilization on cyclic fatigue and torsional fracture resistance of NiTi rotary instruments. Odontology.

[ref-15] Lopes HP, Chiesa WMM, Correia NR, De Souza Navegante NC, Elias CN, Moreira EJL, Chiesa BEC (2011). Influence of curvature location along an artificial canal on cyclic fatigue of a rotary nickel-titanium endodontic instrument. Oral Surgery, Oral Medicine, Oral Pathology, Oral Radiology, and Endodontics.

[ref-16] Martín B, Zelada G, Varela P, Bahillo JG, Magán F, Ahn S, Rodríguez C (2003). Factors influencing the fracture of nickel-titanium rotary instruments. International Endodontic Journal.

[ref-17] Necchi S, Taschieri S, Petrini L, Migliavacca F (2008). Mechanical behaviour of nickel-titanium rotary endodontic instruments in simulated clinical conditions: a computational study. International Endodontic Journal.

[ref-18] Özyürek T, Yılmaz K, Uslu G (2017). The effects of autoclave sterilization on the cyclic fatigue resistance of ProTaper Universal, ProTaper Next, and ProTaper Gold nickel-titanium instruments. Restorative Dentistry & Endodontics.

[ref-19] Pedullà E, Plotino G, Grande NM, Scibilia M, Pappalardo A, Malagnino VA, Rapisarda E (2014). Influence of rotational speed on the cyclic fatigue of Mtwo instruments. International Endodontic Journal.

[ref-20] Pérez-Higueras JJ, Arias A, De la Macorra JC (2013). Cyclic fatigue resistance of K3, K3XF, and twisted file nickel-titanium files under continuous rotation or reciprocating motion. Journal of Endodontics.

[ref-21] Phumpatrakom P, Klinsontorn N, Jutrakulkeeree T, Thanarojwongsa V, Klaisiri A, Aguilar P (2022). Effect of autoclave sterilization on cyclic fatigue of EdgeTaper platinum and ProTaper gold nickel–titanium rotary instruments: an *in vitro* study. Saudi Endodontic Journal.

[ref-22] Plotino G, Costanzo A, Grande NM, Petrovic R, Testarelli L, Gambarini G (2012). Experimental evaluation on the influence of autoclave sterilization on the cyclic fatigue of new nickel-titanium rotary instruments. Journal of Endodontics.

[ref-23] Plotino G, Grande NM, Cordaro M, Testarelli L, Gambarini G (2009). A review of cyclic fatigue testing of nickel–titanium rotary instruments. Journal of Endodontics.

[ref-24] Riyahi AM, Bashiri A, Alshahrani K, Alshahrani S, Alamri HM, Al-Sudani D (2020). Cyclic fatigue comparison of TruNatomy, twisted file, and ProTaper next rotary systems. International Journal of Dentistry.

[ref-25] Setzer FC, Böhme CP (2013). Influence of combined cyclic fatigue and torsional stress on the fracture point of nickel-titanium rotary instruments. Journal of Endodontics.

[ref-26] Shen Y, Haapasalo M, Cheung GS, Peng B (2009). Defects in nickel-titanium instruments after clinical use. Part 1: relationship between observed imperfections and factors leading to such defects in a cohort study. Journal of Endodontics.

[ref-27] Silva EJNL, Giraldes JFN, De Lima CO, Vieira VTL, Elias CN, Antunes HS (2019). Influence of heat treatment on torsional resistance and surface roughness of nickel-titanium instruments. International Endodontic Journal.

[ref-28] Silva EJNL, Zanon M, Hecksher F, Belladonna FG, De Vasconcelos RA, Fidalgo TKDS (2020). Influence of autoclave sterilization procedures on the cyclic fatigue resistance of heat-treated nickel-titanium instruments: a systematic review. Restorative Dentistry & Endodontics.

[ref-29] Siqueira JFJ (2001). Aetiology of root canal treatment failure: why well-treated teeth can fail. International Endodontic Journal.

[ref-30] Venkatesh V, Varghese EJ (2022). Comparative evaluation of cutting efficiency, cyclic fatigue, corrosion resistance, and autoclave cycle effects of three different file systems: an *in-vitro* micro-CT and metallurgy analysis: an original research. Journal of International Oral Health.

[ref-31] Wu J, Lei G, Yan M, Yu Y, Yu J, Zhang G (2011). Instrument separation analysis of multi-used ProTaper Universal rotary system during root canal therapy. Journal of Endodontics.

[ref-32] Yared GM, Bou Dagher FE, Machtou P (2001). Influence of rotational speed, torque and operator’s proficiency on ProFile failures. International Endodontic Journal.

[ref-33] Zhang EW, Cheung GSP, Zheng YF (2010). Influence of cross-sectional design and dimension on mechanical behavior of nickel-titanium instruments under torsion and bending: a numerical analysis. Journal of Endodontics.

